# The influence of socio-economic status on the severity of obstructive sleep apnea: a cross-sectional observational study

**DOI:** 10.5935/1984-0063.20180018

**Published:** 2018

**Authors:** Dimitrios Papadopoulos, Anastasia Kikemeni, Alexandra Skourti, Anastasia Amfilochiou

**Affiliations:** 1 National School of Public Health, Department of Occupational and Industrial Hygiene - Athens - Attica - Grécia.; 2 “Sismanoglio” General Hospital of Attica, Respiratory Function & Sleep Study Unit - Marousi - Attica - Grécia.

**Keywords:** Sleep Apnea, Obstructive, Social Class, Disorders of Excessive Somnolence, Polysomnography

## Abstract

There is limited evidence about the effect of socio-economic status (SES) on the severity of obstructive sleep apnea (OSA). We aimed to investigate this relationship in a referral population in Greece, with regards to other established risk factors. **Methods:** We used a retrospective cross-sectional design to assess socio-economic status based on occupational activity in a sample of 282 OSA patients diagnosed in a public hospital sleep laboratory during one-year period. Demographic, anthropometric and social characteristics, as well as the Epworth sleepiness scale (ESS) scores and apnea-hypopnea indexes (AHI) of subjects in each socio-economic class were recorded and statistically significant differences were explored in univariate and multiple regression analysis. **Results:** 99 (35.1%) of the subjects were categorized in the upper, 70 (24.8%) in the intermediate and 113 (40.1%) in the working class. Subjects of the intermediate class had significantly larger neck circumference than those of the upper class (*p*=0.022). Neither class differed significantly in terms of ESS score and intermediate class had a trend for higher AHI than upper class (*p*=0.075 in univariate and *p*=0.082 in multivariate analysis). Age (*p*=0.020) and occasional alcohol consumption (*p*=0.022) were independent negative and neck circumference (*p*<0.001) positive correlates of the variance in ESS score, while body mass index (*p*=0.004), neck circumference (*p*<0.001), being married (*p*=0.014) and current smoker (*p*=0.025) were independent positive correlates of the variance in AHI. **Discussion:** SES has a minor effect on OSA severity that is only partially accounted for by other known risk factors. Neck circumference was found to be the most useful predictor of both subjective daytime sleepiness and severity of respiratory events during sleep.

## INTRODUCTION

Obstructive sleep apnea (OSA) is a disorder characterized by recurrent episodes of partial or complete obstruction of the upper airway during sleep. Its increasing prevalence is consistent with the frequency of established risk factors, such as older age and obesity^1^. Functional outcomes, such as excessive daytime sleepiness (EDS) and poor concentration, largely reversible with successful treatment, may affect occupational performance^2^ and increase the risk of road traffic^3^ and occupational accidents^4^. On the other hand, occupation and socio-economic status (SES) have been described in recent research as independent prognostic factors for OSA presence^5^, severity^6^, cardiovascular consequences^7^ and adherence to treatment^8^, although controlling for potential confounders remains a matter for consideration.

The objectives of the current study were to investigate the relationship between OSA severity and SES, measured by occupational activity, in a sample referred for sleep study in a public hospital sleep laboratory and to corroborate whether possible differences are explained by other known risk factors, such as age, sex, obesity, smoking and alcohol consumption. We have hypothesized that lower class workers would have greater OSA severity, mainly as a result of their greater exposures to unhealthy habits and behaviors^9^.

## METHODS

A retrospective cross-sectional study was conducted in the setting of a public-hospital-based sleep laboratory in Athens. All referred patients were evaluated by a sleep specialist physician in the outpatient department, where they were asked to complete a questionnaire on demographics, social history, sleep habits, nighttime and daytime symptoms and other health problems, as well as the Greek version of the Epworth sleepiness scale (ESS)^10^.

Those judged as having high probability of OSA underwent a diagnostic polysomnography (PSG) with a digital recording system (Alice 3, 4 and 5 Diagnostic Sleep System, Philips Respironics) that monitors the following variables: electroencephalogram (two paracentral, two frontal and two occipital leads), right and left electrooculograms, submental and anterior tibial electromyograms, electrocardiogram, nasal (nasal pressure transducer) and oronasal airflow (thermistor), respiratory effort (thoracoabdominal respiratory inductance plethysmography belts) and pulse oximetry.

Sleep and respiratory event scoring was done manually in 30-second epochs according to recommendations by American Academy of Sleep Medicine^11^. An apnea was scored when a reduction in the oronasal flow signal by ≥90% from baseline was recorded for at least 10s. Apneas were classified as obstructive or mixed in the presence of inspiratory effort in the entire event or a part of it respectively. Hypopnea was scored when a reduction in the nasal pressure signal by ≥30% from baseline was recorded for at least 10s, accompanied with a ≥3% oxygen desaturation from baseline or an arousal. The study protocol was approved by the Ethics Committee of the National School of Public Health.

The records of all patients who underwent diagnostic PSG between January 1 and December 31, 2015, were searched and analyzed. Titration, follow-up and studies with total sleep time less than 2 hours were excluded. The diagnosis of OSA was made according to the diagnostic criteria in the third edition of the International Classification of Sleep Disorders^12^, that require the presence of 15 or more predominantly obstructive respiratory events per hour of sleep or the combination of 5 or more obstructive events per hour with appropriate symptoms and comorbid conditions. Symptomatology and comorbidities were assessed in each patient by their answers in the questionnaire. OSA severity was evaluated on the basis of the following parameters: frequency of respiratory events with calculation of the apnea-hypopnea index (AHI) and degree of subjective daytime sleepiness with ESS score.

The European Socio-economic Classification (ESeC) was used as an indicator of SES. It is a categorical social stratification scheme based on the idea that in market economies, the market position and in particular the occupational division of labor is fundamental to the production of social inequalities. Classification is performed according to occupation, employment status (employer, manager, supervisor, employee, self-employed) and the size of the organization^13^.

Subjects were first given a 3-digit code, categorizing them in minor occupational groups of the International Standard Classification of Occupations (ISCO-88)^14^ based on their response about their current or last professional activity in the questionnaire. This code was used to derive the ESeC ranking under the simplified method, since information about employment status and size of organization was unavailable, which has approximately 80% agreement with the full model. The classes were then collapsed in the 3-class version, “salariat”, “intermediate” and “working class”, for analysis purposes. Housewives and students were excluded from statistical analysis, since they are not actively seeking work and their SES is influenced by the occupation and income of other family members.

Variables that were considered potential confounders included sex, age, anthropometric characteristics, such as body mass index (BMI), neck circumference and waist to hip ratio (WHR), marital status, smoking, alcohol consumption and years of education. The anthropometric data were measured by the unit staff before PSG, while the rest were self-reported.

In descriptive statistical analysis, continuous variables were expressed as mean ± standard deviation and categorical in the form of frequencies. Where continuous variables were not normally distributed the median and interquartile range (IQR) were also given. Frequency differences between categorical variables were analyzed by the chi-square test, while the differences in means between continuous variables with analysis of variance (ANOVA) or Kruskal-Wallis test, depending on the normality of data. For post hoc analysis we applied the Bonferroni adjustment for pairwise comparisons to account for type-1 errors.

To examine associations between several variables we used multiple linear regression models with the backward stepwise method. Non-normally distributed dependent variables were transformed to their square roots before performing linear regression, while categorical independent variables were entered in the regression model in the form of dummy variables. We also implemented ordinal regression to examine the probabilities of independent variables to predict a higher severity category of AHI, applying the usual cut-offs of 15 and 30 events/h to distinguish between mild, moderate and severe OSA^12^.

Missing data were handled with multiple imputation, assuming they were missing at random, using the fully conditional specification method. Suitable transformations were used to deal with non-normality and then complete data were transformed back to their original scales before analysis. Five sets of data were created and the results of linear or ordinal regression in each set were pooled to report an average estimate.

All significance tests were two-sided, with *p*-value < 0.05 being considered statistically significant. Analysis was conducted with the statistical software package IBM^(®)^ SPSS^(®)^ Statistics Version 20.

## RESULTS

Among 399 patients who underwent PSG during the study period, 53 were excluded for not fulfilling the inclusion criteria and 36 for not having an OSA diagnosis. From the rest, we could not codify 28 patients in neither of the 3 socio-economic classes (housewives=20, students=2, long term unemployed=1, insufficient information=5), limiting the final sample to 282 patients. 241 were males (85.5%) and 41 females (14.5%), while the mean age of the sample was 54.61±12.17. The distribution of subjects in socio-economic classes and the demographic, anthropometric and social characteristics of the sample per class are presented in Table 1. Subjects of the intermediate class had significantly larger neck circumference than those of the upper class (*p*=0.022). As expected, salariat and working class had more and less years of education respectively than intermediate class (*p*<0.001). There were no significant differences between the 3 classes in terms of symptoms and associated diseases, except from the fact that individuals of the upper class had significantly more complaints of non-restorative sleep than the other groups (chi-square 10.561, *p*=0.005).

**Table 1:** Descriptive statistics of study population per socio-economic class with missing data frequencies.

Variable	EseC 1 (Ν=99)	EseC 2 (Ν=70)	EseC 3 (Ν=113)	*p*
Sex				
*Male*	84 (84.8)	63 (90)	94 (83.2)	0.436
*Female*	15 (15.2)	7 (10)	19 (16.8)	
*Age (years)*	54.44±12.33	55.43±12.05	54.25±12.18	0.806
*BMI (kg/m^2^)*	33.43±5.93	35.41±6.88	34.68±6.62	0.127
Missing data	0 (0)	1 (1.4)	0 (0)	0.219
*Neck circumference (cm)*	42.26±3.48	43.61±3.59	42.95±3.67	0.025*
Median (IQR)	42 (40-44)	43 (41-47)	43 (41-46)	
Missing data	0 (0)	1 (1.4)	2 (1.8)	0.430
*WHR*	1±0.1	1.01±0.05	1.01±0.08	0.376
Missing data	0 (0)	1 (1.4)	2 (1.8)	0.430
Marital status				
*Married*	75 (75.8)	54 (77.1)	97 (85.8)	0.336
*Single*	21 (21.2)	13 (18.6)	16 (14.2)	
Missing data	3 (3)	3 (4.3)	0 (0)	0.110
Smoking				
*Smoker*	34 (34.3)	28 (40)	49 (43.4)	0.314
*Former smoker*	32 (32.3)	28 (40)	35 (31)	
*Non-smoker*	32 (32.3)	14 (20)	28 (24.8)	
Missing data	1 (1)	0 (0)	1 (0.9)	0.713
Alcohol consumption				
*Daily*	4 (4)	11 (15.7)	13 (11.5)	0.053
*Occasionally*	45 (45.5)	20 (28.6)	43 (38.1)	
*Almost never*	47 (47.5)	36 (51.4)	55 (48.7)	
Missing data	3 (3)	3 (4.3)	2 (1.8)	0.603
Years of education				
*<7*	0 (0)	3 (4.3)	18 (15.9)	<0.001
*7-12*	5 (5.1)	20 (28.6)	54 (47.8)	
*>12*	88 (88.9)	39 (55.7)	23 (20.4)	
Missing data	6 (6.1)	8 (11.4)	18 (15.9)	0.078

BMI=body mass index; WHR=waist to hip ratio; IQR=interquartile range

* statistical significant pairwise comparison only between EsEC 1 and EsEC 2 (*p*=0.022)

Categorical and missing data are presented as frequency count (%) and analyzed with chi-square test Continuous data are presented as mean ± standard deviation and analyzed with one-way ANOVA, except from neck circumference presented also as median (IQR) and analyzed with Kruskal-Wallis test

The mean AHI of the sample was 43.32±27.1 and 59.9% of the subjects had severe OSA, based on an AHI ≥30. The mean ESS score was 9.16±5.16. There was a statistically significant positive, but weak, correlation between AHI and ESS score (Spearman’s rho 0.236, *p*<0.001). The mean and median AHI and ESS scores and the frequencies of the severity categories across socio-economic classes are shown in Table 2. Intermediate class was found to have the highest AHI, although the overall difference between classes was marginally non-significant (*p*=0.059). In post-hoc analysis the above trend was only observed between higher and intermediate class (*p*=0.075).

**Table 2:** Mean AHI and ESS scores and prevalence of AHI severity categories of study population per socio-economic class with missing data frequencies.

Outcome variable	ESeC 1	ESeC 2	ESeC 3	*p*
*ESS score*	9.24±5.27	9.41±4.9	8.92±5.2	0.645
Median (IQR)	8.5 (5-12)	9 (6-13)	8 (5-12)	
Missing data	1 (1)	2 (2.9)	4 (3.5)	0.485
*AHI*	39.81±27.01	50.51±30.43	41.94±24.26	0.059
Median (IQR)	31.7 (16.7-63.5)	45 (24-78.6)	39.3 (22-64.4)	
AHI groups				
*<15*	23 (23.2)	6 (8.6)	18 (15.9)	0.133
*15-29.9*	23 (23.2)	19 (27.1)	24 (21.2)	
*≥30*	53 (53.5)	45 (64.3)	71 (62.8)	

ESS=Epworth sleepiness scale; AHI=apnea-hypopnea index; IQR=interquartile range Categorical and missing data are presented as frequency count (%) and analyzed with chi-square test Continuous data are presented as mean ± standard deviation, median (IQR) and analyzed with Kruskal-Wallis test

A multiple linear regression analysis was performed to reveal statistically significant predictors of the variance in ESS score and AHI (Table 3). To achieve normal distribution, both variables were transformed to their square roots before analysis. Because of the linear association between socio-economic status and years of education, the latter variable was excluded from analysis to avoid multicollinearity.

**Table 3:** Pooled estimated results of multiple linear regression analysis with the backward stepwise method for the dependent variables √ESS and √AHI after multiple imputation of missing data.

Independent variable	B Coefficient (SE)	*p*
	*Dependent variable: √ESS*	
Age (per year increase)	-0.01 (0.004)	0.020
Neck circumference (per cm increase)	0.06 (0.016)	<0.001
WHR (per unit increase)	-1.33 (0.718)	0.065
Alcohol consumption (vs almost never)		
*Daily*	-0.34 (0.174)	0.051
*Occasionally*	-0.26 (0.111)	0.022
	*Dependent variable: √AHI*	
BMI (per unit increase)	0.06 (0.021)	0.004
Neck circumference (per cm increase)	0.2 (0.039)	<0.001
Marital status (married vs single)	0.74 (0.299)	0.014
Smoking (smoker vs non-smoker)	0.51 (0.228)	0.025
Socio-economic class (intermediate vs salariat)	0.45 (0.257)	0.082

ESS=Epworth sleepiness scale; AHI=apnea-hypopnea index; BMI=body mass index; WHR=waist to hip ratio Variables entered: sex, age, BMI, neck circumference, WHR, marital status, smoking (two dummy variables), alcohol consumption (two dummy variables), socio-economic class (two dummy variables) Criterion for stepwise variable removal: *p*>0.1

For √ESS, age and occasional alcohol consumption were significant negative correlates and neck circumference was significant positive correlate, explaining the variance in ESS score by 10%. Respectively, BMI, neck circumference, being married and current smoker were independent positive correlates for √AHI, the model accounting for 28% of the variance. SES was not an independent predictor in both models (*p*>0.05), using salariat as the reference category. In the √AHI model, however, the significance of the difference between higher and intermediate class remained over the 90% confidence level (*p*=0.082).

We further performed an ordinal logistic regression analysis in order to reveal significant predictors of OSA severity according to AHI category (Table 4). Since the frequency counts constantly increase from the lowest to the highest severity category in our sample, we used the complementary log-log link function to transform the cumulative probabilities. In this model, only neck circumference was statistically significant (*p*=0.006), predicting a 14% increase in the odds of being in a higher severity category for every 1 cm increase.

**Table 4:** Pooled estimated results of ordinal logistic regression analysis with the complementary log-log link function for the AHI dependent variable ordered by severity category after multiple imputation of missing data.

Independent variable	OR (CI 95%)	*p*
Sex		
*Male*	1.54 (0.75-3.20)	0.243
*Female*	1*	
Age (per year increase)	1.01 (0.99-1.03)	0.256
BMI (per unit increase)	1.04 (0.99-1.09)	0.089
Neck circumference (per cm increase)	1.14 (1.04-1.24)	0.006
WHR (per unit increase)	0.36 (0.03-4.63)	0.434
Marital status		
*Married*	1.41 (0.88-2.27)	0.150
*Single*	1*	
Smoking		
*Smoker*	1.34 (0.81-2.21)	0.252
*Former smoker*	0.86 (0.53-1.40)	0.547
*Non-smoker*	1*	
Alcohol consumption		
*Daily*	0.79 (0.40-1.53)	0.483
*Occasionally*	0.92 (0.58-1.46)	0.714
*Almost never*	1*	
Socio-economic class		
*Salariat*	1*	
*Intermediate*	1.15 (0.69-1.93)	0.588
*Working class*	1.16 (0.75-1.79)	0.500

BMI=body mass index; WHR=waist to hip ratio

* Reference category.

## DISCUSSION

Our initial hypothesis that OSA patients from low socio-economic classes would present with greater severity of respiratory events during sleep and subjective daytime sleepiness was not confirmed. There seems to be a trend for higher AHI in intermediate class, although it did not reach statistical significance neither in univariate nor in multivariate analysis. Moreover, intermediate class in our sample had significant higher neck circumference than upper class and that difference could partially be responsible for the observed trend. The social factors that were found to independently predict the variance in OSA severity were marital status and smoking for AHI variance and alcohol consumption for ESS score variance.

The relationship between low SES and obesity^15^, increased consumption of tobacco^16^ and alcohol^17^, consistently found in literature, was not observed in our sample. In fact, our data show that intermediate class have more similarities in obesity scores and patterns of social habits with working class than salariat, implying that they could also share similar health risks. The last decade’s economic crisis in Greece has mostly affected the urban middle class, shrinking its income and widening the gap between the wealthier upper classes and the lower ones, resulting in lower self-rated health^18^ and rising unmet needs for health care^19^.

We did not find a statistically significant effect of SES on OSA severity, assessed by ESS score and AHI, after applying multiple regression models to control for potential confounders, such as age, sex, body habitus measurements and social factors. Ramsey et al. studied 4042 OSA patients and comparing income categories in terms of AHI found also no significant differences after adjustment for BMI in both sexes^6^.

However, the trend for higher AHI in subjects of the intermediate class compared with upper class, which was included in our final multiple regression model for AHI variance, even with lower level of statistical significance (*p*<0.1), cannot be attributed solely to anthropometric differences. Possible explanations are differences in referral patterns between classes, since upper class often has better access to health care, or it could reflect differences between distinct occupations in each class. The majority of subjects of the intermediate class in our sample were office clerks (52.9%), being at most a sedentary occupation. In recent literature, light activity or sedentary occupations have been associated with increased risk for moderate to severe OSA^20^.

Despite non-significant differences in AHI and ESS score between classes, patients of the upper class complained significantly more for not obtaining restorative sleep most of the nights than the other classes. It is possible that, since they are more educated and thereby more cultured, they would recognize easier their symptoms and their day-to-day variability.Results of a large US cross-sectional epidemiologic survey also showed that individuals with the lowest educational attainment, particularly immigrants, reported fewer sleep symptoms than the more educated groups or the native born^21^. In a similar Brazilian survey, subjects with higher family income were more likely to report the presence of any sleep complaint, as well as insufficient sleep, snoring and bruxism, while those with lower income complained more about insomnia and superficial sleep^22^.

Older age and social alcohol drinking were protective factors for subjective daytime sleepiness in our multiple regression model. Previous studies have reproduced the same findings, using both subjective and objective measurements of EDS. Bixler et al.^23^ examined a large Pennsylvanian cohort from the general population and observed that increasing age was associated with less subjective EDS, suggesting the presence of unsatisfied sleep needs and depression in the young. Budhiraja et al.^24^ recently showed that ESS score decreased and mean sleep latency in maintenance of wakefulness test increased with advancing age in a multicenter OSA cohort, giving the explanation of disrupted sleep homeostatic mechanisms with ageing. However, since elderly individuals often consider their sleepiness normal and EDS was found to have no impact on quality of life of elderly OSA patients^25^, it is also possible that they seek less frequently medical assistance than younger sleepy OSA patients. 

Regarding alcohol consumption, Pack et al.^26^ found in a sample of older adults that alcohol use reduced the risk for subjective EDS, hypothesizing that awareness of the negative effect of alcohol on sleep gradually leads to a decrease in its consumption. A similar result was obtained from a population survey in US^27^; however, the authors discovered that the interaction between heavy alcohol drinking and decreased sleep duration predicted increased EDS and considered sleep duration to be a confounding factor. Despite the objectively evaluated detrimental effects of alcohol consumption on sleep and daytime alertness in multiple studies, alcohol users may still perceive its impact as beneficial and rate it accordingly, perhaps due to differential expectations^28^. Further research using both subjective and objective measurements of EDS is required to test this assumption in OSA patients. The fact that these risk factors account for only a small percentage of ESS score variance in our sample highlights the multifactorial nature of EDS.

Obesity, large neck circumference and smoking were independent risk factors for higher AHI in our study, results consistent with previous research. Peppard et al.^29^ showed that a 10% weight gain in 4 years predicted 32% increase in AHI. Neck circumference has been recognized as better predictor of OSA severity than visceral obesity, especially in non-obese patients^30^, and smoking has been associated with upper airway inflammation and narrowing, worsening OSA^31^. The finding that married patients had significantly higher AHI than singles was also observed in another clinical-based study but not in community samples^32^. Since referral patterns between married and unmarried patients can substantially differ, depending on the presence of a bed partner who witnesses the relevant symptoms and behaviors, this relationship can be subjected to selection bias rather than represent a true association. Moreover, the finding from our ordinal regression model that neck circumference was the only significant correlate of the probability of being in a higher severity category in terms of AHI implies that, unlike the social factors examined, it could serve as a useful predictor in clinical practice, being able to identify the most severe OSA cases.

Our study has several limitations. Because of the retrospective nature of our data, classification in socio-economic classes was based in a single open-type question about subjects’ most recent occupational activity. As a result, the entirety of description varied greatly between individuals, allowing us to codify some of them in hierarchically less detailed occupational group (major or sub-major) in ISCO-88. It is, however, possible that this simplification could in some cases overestimate or underestimate the positioning in socio-economic class in relation to subjects’ actual occupation. In the same manner, there were no information about length of employment and former occupational activities.

Since subjects in this study had not emerged from sampling of the general population, where patients with less severe OSA are more likely to exist, caution must be taken when attempting to generalise our results to the whole referent population. Furthermore, the cross-sectional design lacks definite power in finding causative associations between outcome and exposure, because they were assessed at the same time.

In conclusion, we have shown that SES has a minor effect on OSA severity. Intermediate class patients tend to have worse OSA than upper class, although differences in certain obesity indices were also noted. Further research with prospective studies is required to test the effect of SES on OSA presence and severity. Already known risk factors, such as obesity, large neck circumference and smoking, were found independent predictors of severity of respiratory events at sleep, while the role of alcohol consumption and marital status on OSA severity needs further clarification in future research.
